# X-ray structure of *Fasciola hepatica* Sigma class glutathione transferase 1 reveals a disulfide bond to support stability in gastro-intestinal environment

**DOI:** 10.1038/s41598-018-37531-5

**Published:** 2019-01-29

**Authors:** Kirsty Line, Michail N. Isupov, E. James LaCourse, David J. Cutress, Russell M. Morphew, Peter M. Brophy, Jennifer A. Littlechild

**Affiliations:** 1 Henry Wellcome Building for Biocatalysis, Biosciences, College of Life and Environmental Sciences, University of Exeter, Stocker Road, Exeter, EX4 4QD UK; 2 Institute of Biological, Environmental and Rural Sciences (IBERS), Aberystwyth University, Aberystwyth, SY23 3DA UK; 3 Liverpool School of Tropical Medicine, Liverpool, Pembroke Place, L3 5QA UK

## Abstract

Sigma class GST (Prostaglandin D synthase), FhGST-S1, is present in the excretory–secretory products (ES) of the liver fluke parasite *Fasciola hepatica* as cargo of extracellular vesicles (EVs) released by the parasite. FhGST-S1 has a well characterised role in the modulation of the immune response; a key fluke intercession that allows for establishment and development within their hosts. We have resolved the three-dimensional structure of FhGST-S1 in complex with its co-factor glutathione, in complex with a glutathione-cysteine adduct, and in a glutathione disulfide complex in order to initiate a research pipeline to mechanistically understand how FhGST-S1 functions within the host environment and to rationally design selective inhibitors. The overall fold of FhGST-S1 shows high structural similarity to other Sigma class GSTs. However, a unique interdomain disulfide bond was found in the FhGST-S1 which could stabilise the structure within the host gastro-intestinal environment. The position of the two domains of the protein with respect to each other is seen to be crucial in the formation of the active site cleft of the enzyme. The interdomain disulfide bond raises the possibility of oxidative regulation of the active site of this GST protein.

## Introduction

The digenean parasitic worms *Fasciola hepatica* (temperate liver fluke) and *Fasciola gigantica* (tropical fluke) are responsible for Fasciolosis. This neglected disease has a significant impact on global food security by causing economic losses of over US$ 3 billion per annum to livestock through mortality, reduction in host fecundity, susceptibility to other infections, decrease in milk, meat production and condemnation of livers^1^. The impact on the livestock industry is most likely underestimated as the immunomodulatory activity of liver fluke complicates control measures of additional livestock diseases such as bovine tuberculosis^2^.

Fasciolosis is re-emerging as a food borne disease in human populations with outbreaks in Bolivia, Peru, Ecuador, Vietnam, Thailand, Egypt and Iran^1,3,4^. Animals and humans become infected by ingesting vegetation or water contaminated with the infective larval stage of the parasite, the metacercariae. Following ingestion, metacercariae excyst from their dormant stage and penetrate the intestinal wall before migrating to the liver and subsequently the bile ducts where they develop into their mature form^5^. Liver fluke control, in the continued absence of protective vaccines, is dominated by chemotherapy. Triclabendazole (TCBZ) is the drug of choice for humans and domesticated animals as it targets both pathogenic juvenile and adult fluke. TCBZ failure and resistance is increasingly being reported in many countries, raising concerns of how to control the disease in the absence of new flukicides and vaccines^6^.

The GST (EC 2.5.1.18) soluble superfamily forms a large and diverse enzyme group with at least seven species independent classes identified; namely Alpha, Theta, Zeta, Mu, Pi, Sigma and Omega and other species or phyla specific families^7^. The primary enzymatic role of GST is the Phase II drug metabolism conjugation of reduced glutathione (GSH) to a wide range of hydrophobic exogenous and endogenous toxic compounds resulting in their inactivation and an increase in their water solubility for ease of excretion and further metabolism^8^. The GSTs also have predicted roles in sequestration of toxins as part of Phase III drug metabolism^8^. In adult helminth parasites, GSTs are potentially the major detoxification system, with limited Phase I cytochrome P450 activities and limited other Phase II activities^8,9^.

GSTs were presumed to be only detoxification proteins, however house-keeping activities in metabolism have been identified. For example, Sigma GSTs have been found to have prostaglandin synthase activity and other GSTs have been shown to be involved in the binding of a number of hydrophobic substrate compounds for transport^9^. Structures of GST family enzymes have revealed a conserved canonical GST fold with two domains. The smaller N-terminal domain consists of a thioredoxin like fold, whilst the larger C-terminal domain is entirely α-helical^7^.

Parasitic helminths may have adapted the house-keeping role of Sigma GSTs in synthesising prostaglandins to help modulate the immune response of their hosts. In *F. hepatica* a new Sigma class GST (FhGST-S1) was identified by proteomic analysis^10^ and confirmed to be an excretory–secretory protein^11^ that can be transported from the parasite via extracellular vesicles^12^. Purified FhGST-S1 was confirmed to possess prostaglandin synthase (PGDS) activity and the enzyme activated host immune cells (dendritic cells and macrophages) to produce prostaglandins and elevated levels of Th2 cytokines. Thus, extracellular FhGST-S1 manipulations of host cell phenotypes may support *F. hepatica* to drive the Th2 response and establish its long-term infections^11,13^. Furthermore, FhGST-S1 when trialled as a vaccine reduced pathology normally associated with liver fluke infection^14^.

Structural information is a prerequisite for mechanistically understanding biochemical activity and for the rational design of inhibitor compounds. Here we present the 1.6 Å three-dimensional structures of the FhGST-S1 in the presence of GSH alone (holo-structure), cysteine covalently attached to the GSH (CGL complex), and of a glutathione disulfide (GSSG-structure).

## Results and Discussion

### Structural studies

Both the FhGST-S1 holoenzyme and the GSSG complex were crystallised in the same orthorhombic space group with slightly different unit cells (Table 1). As the crystallisation media contained potassium bromide several bromide binding sites with partial occupancy were located in both structures. Several DMSO molecules were located in the GSSG complex structure, where this organic solvent was added to increase the solubility of the inhibitor TCBZ. The electron density obtained from the structural results has allowed the positioning of 209–210 amino acid residues out of the 211 in each monomer in both structures. The electron density was of high enough quality to differentiate several main chain second positions and multiple alternative side chain positions in both structures. The disulfides in both structures were partially formed and the cysteines were refined both as disulfide and free thiols. In both structures Pro54 of each subunit is in the *cis* conformation. Both models have acceptable stereochemical parameters for their observed resolution as judged by PROCHECK^15^. Of the non-glycine residues in the protein 92% fall into the most favoured regions of the Ramachandran plot for both the holo and GSSG complex structures.

**Table 1:** Summary of data processing and model refinement statistics.

	Holoenzyme	GSSG complex
Space group	P2_1_2_1_2_1_	P2_1_2_1_2_1_
Unit cell parameters a, b, c (Å)	56.7, 87.8, 93.8	53.9, 87.4, 94.0
Resolution Range (Å)	19.43-1.59	20.00-1.61
Completeness (%)	98.2 [91.9]^a^	95.5 [77.9]^a^
*R*_sym_	0.074 [0.485]^a^	0.072 [0.59]^a^
< I/ σI >	13.7 [1.7]^a^	16.1 [1.7]^a^
Redundancy	3.8	4.6
Unique reflections	62227	56342
*B*-factor of data from Wilson plot (Å^2^)	23.2	22.2
Final *R*_cryst_ (%)	17.24	17.80
*R*_free_ (2.0% total data: %)	21.61	21.67
No. of protein residues	419	419
Average *B*-factor (protein: Å^2^)	22.8	22.3
No. water molecules	624	612
Average *B*-factor (water: Å^2^)	36.7	35.2
Average *B*-factor (ligands: Å^2^)	18.6	23.1
Rms deviations from ideality^a^		
Bond lengths (Å)	0.009 (0.022)^b^	0.010 (0.019)^b^
Bond angles (°)	1.344 (1.998)^b^	1.399 (2.011)^b^
Ramachandran plot analysis (% of residues)		
Most favoured	92.5	93.6
Additionally allowed	6.6	5.8
Generously allowed	0.8	0.6
Disallowed	0	0
G-factor	0.2	0.1

^a^Values for the outer resolution shell are given in brackets.

^b^Target values are given in parentheses. R_sym_ = ∑_h_∑_J_| < I_h_ > −I _J_(h) |/∑_h_∑_J_I(h), where I(h) is the intensity of the reflections h, ∑_h_ is the sum over all t_h_e reflections and ∑_J_ is the sum over J measurements of the reflections. R_cryst_ = ∑||Fo|−|Fc||/∑|Fo|. Wilson B-factor was estimated by SFCHECK^36^. The Ramachandran plot analysis and G-factor calculation were performed by PROCHECK^15^.

### Monomer structure

**Figure 1:**
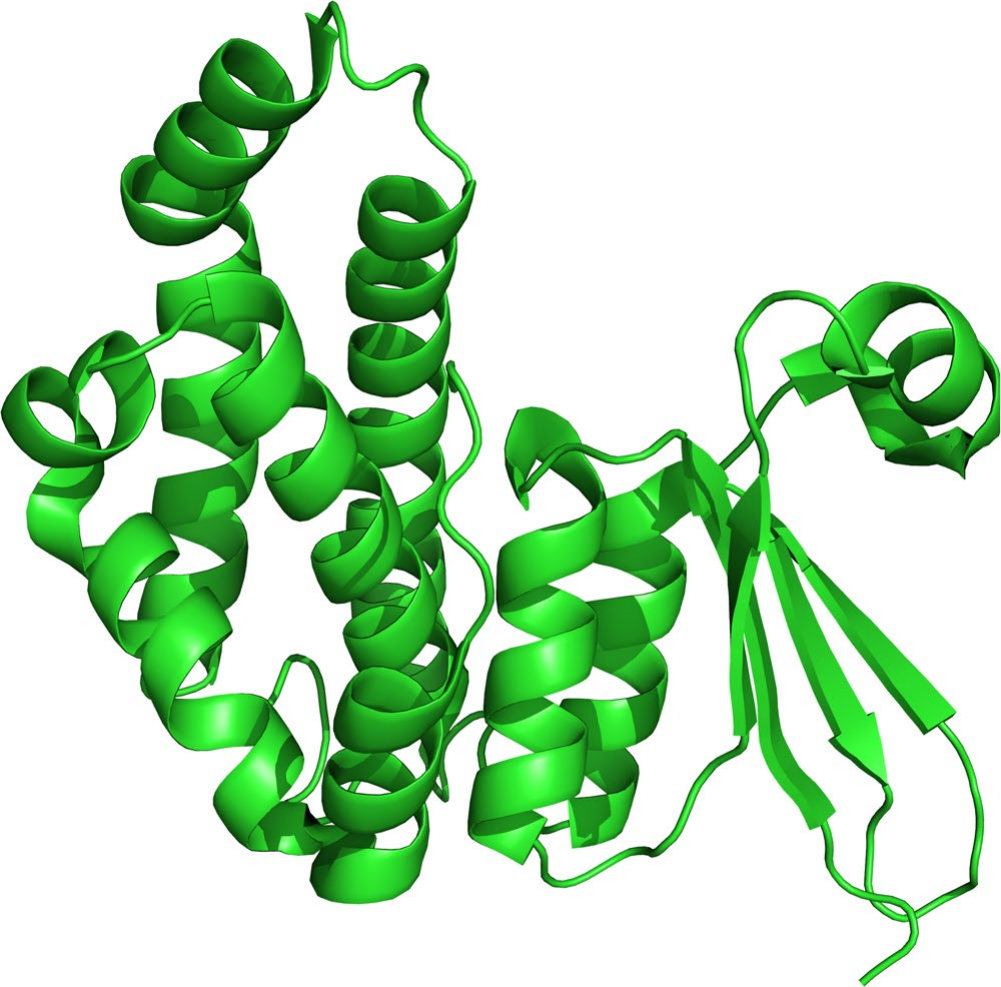
A cartoon presentation of the monomer fold of FhGST-S1.

**Figure 2:**
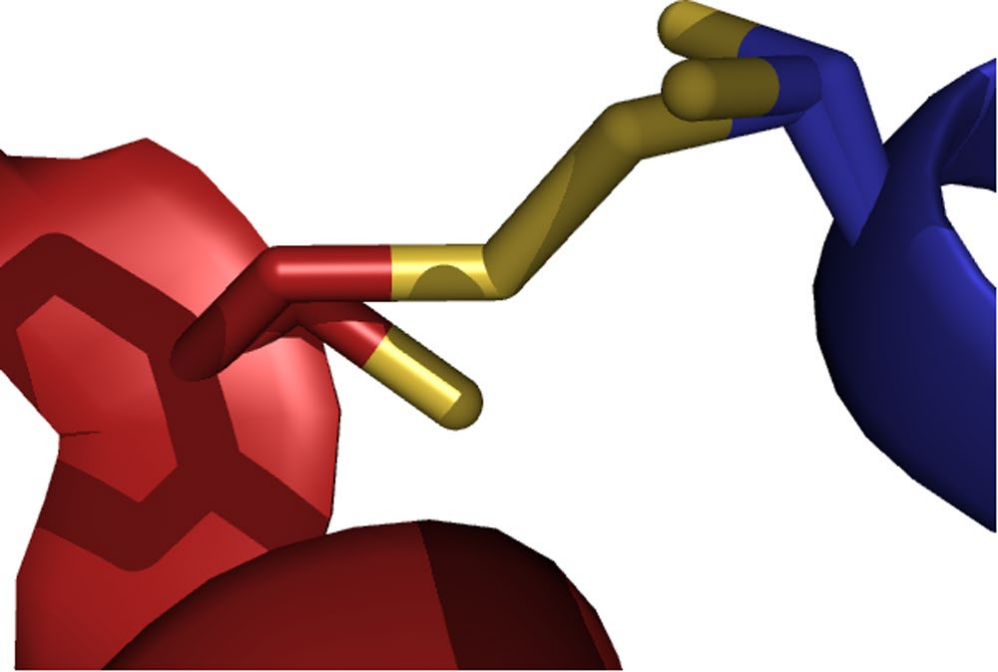
The interdomain disulfide bond shown as a link between the Sγ atoms (yellow) of the two cysteine residues, which has partial occupancy. Alternative conformations of the side chains of cysteine residues 26 and 196 are shown as stick models. Secondary structure elements and carbon atoms of the N-terminal domain are shown in blue and of the C-terminal domain in red.

The FhGST-S1 monomer adopts the canonical GST fold with two domains, the smaller N-terminal domain with a thioredoxin-like fold (residues 2–83) and the larger all α-helix C-terminal domain (residues 84–211) (Fig. 1). The N-terminal domain is formed by a 4 stranded mixed β-sheet with Richardson topology −1 × 2 1^16^. The sheet is flanked by helices α1 (residues 15–26) and α3 (residues 71–81) on one side and on the other by α2 (residues 41–51) including the short 3_10_ helices α2 and α2′. The N-terminal domain has the folding pattern βαβαββα. Residue Pro54 at the loop between helix α2′ and strand β3 is in the *cis* conformation. Conservation of a *cis* residue at this position has been reported for all GST structures^17^. A β-bulge is present after strand β3 (residue Gly 60). The all α-helical C-terminal domain contains 6 helices. The N- and C-terminal domains interact through packing of helices α1 and α3 against helices α6 and α4 with interactions of helix α9 with helix α1. Each monomer contains an intermolecular disulfide bond between cysteine residues C26 and C196 at partial occupancy (Fig. 2). This results in a bond between the N-terminal domain helix α1 and the C-terminal domain helix α9. This is the first time that such an interdomain disulfide bond has been observed in a GST. The active site is located in the cleft between the two domains of the monomer and as such would be altered by movement between the two domains. The presence of the interdomain disulfide bond raises the possibility of oxidative regulation of the active site of this GST protein.

### Quaternary Structure

The FhGST-S1 forms dimers related by a molecular dyad with approximate dimensions 46 Å × 53 Å × 50 Å. The formation of the dimer buries 2922 Å^2^ of the solvent accessible surface area. This means that 13% of the subunit solvent accessible surface of 11189 Å^2^ is buried upon oligomerization. Interaction between the two subunits is by 11 hydrogen bonds and 151 non-bonded contacts between 21 amino acid residues from each chain. Like other sigma class GSTs, FhGST-S1 lacks the hydrophobic “lock and key” motif found in Alpha, Mu and Pi class GSTs^18^.

### Active Site

**Figure 3:**
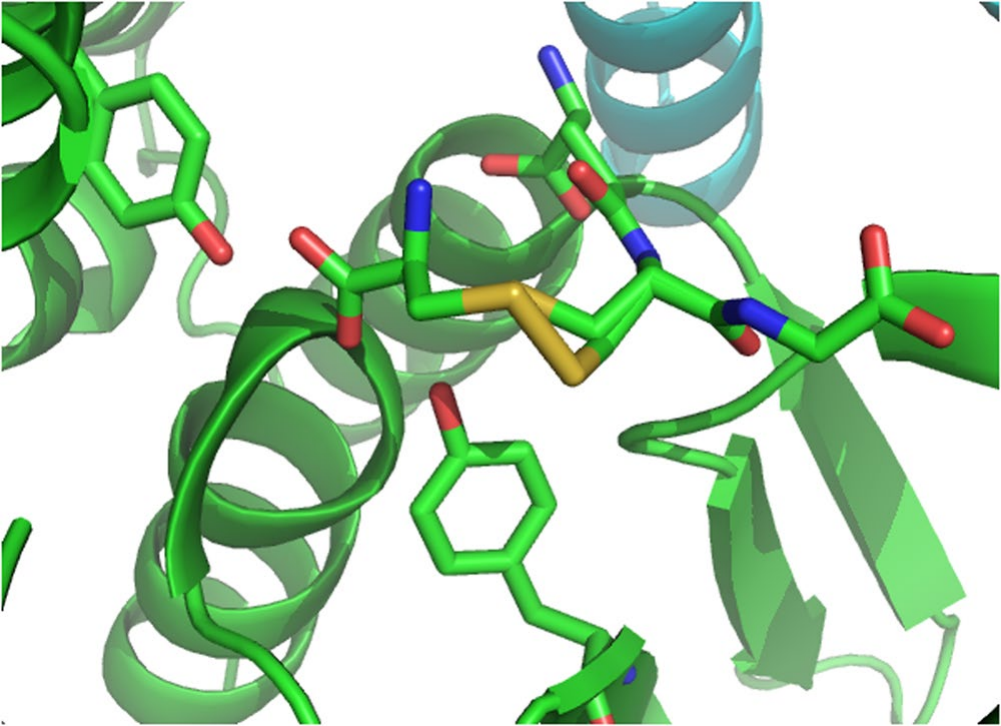
The G-site is located on the interface of two FhGST-S1 GST monomers, which are shown as a cartoon representation in colours green and cyan respectively. The CGL complex, which contains GSH forming a disulfide bond with a free cysteine residue, is shown bound to the G-site. The CGL molecule and the two active site tyrosine residues, 10 and 106 are presented as stick models.

**Figure 4:**
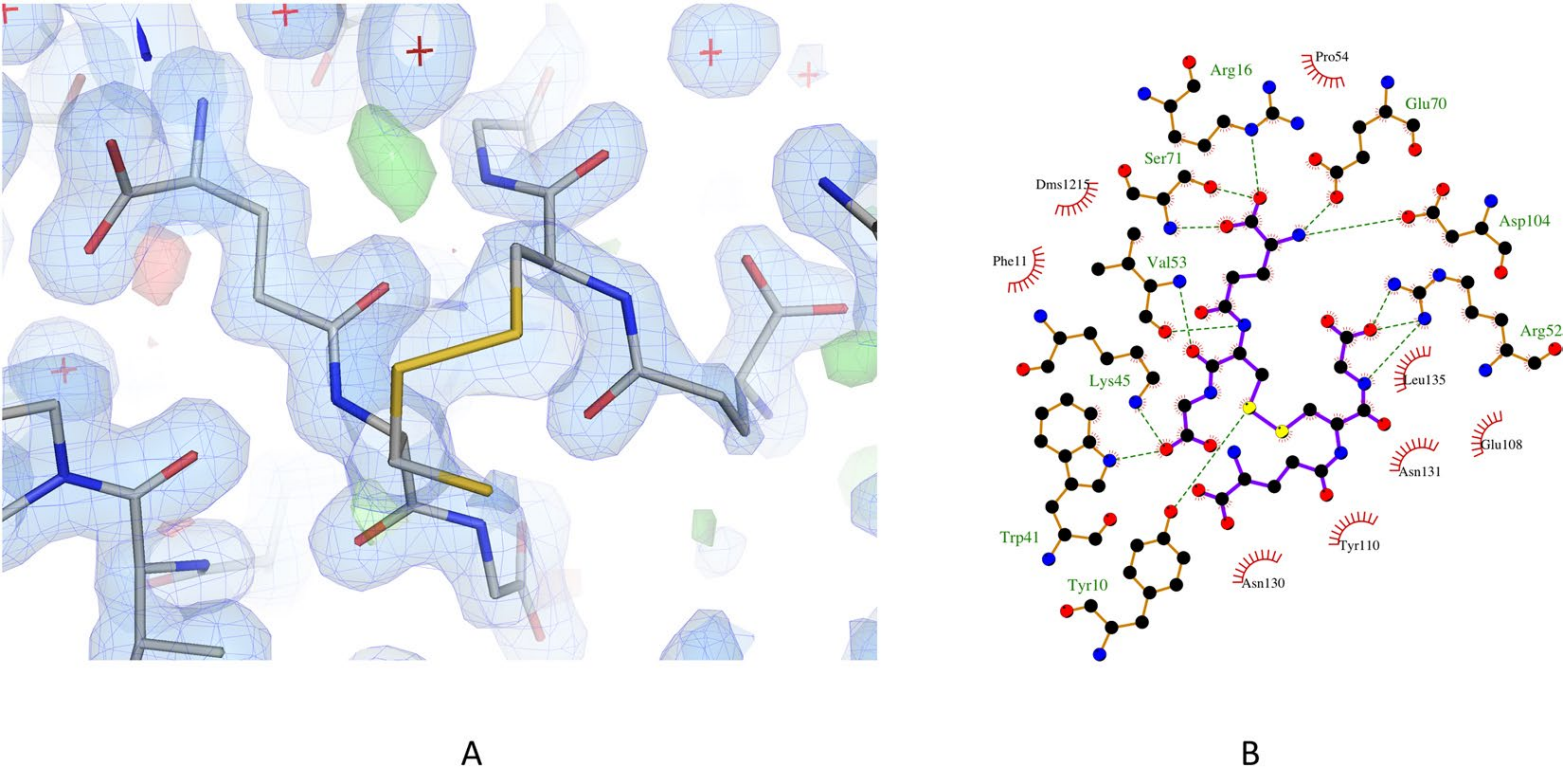
The GSSG complex interactions in the active site of FhGST-S1. (**a**) Electron-density maps of the active site of the FhGST-S1 GSSG complex. The 2Fo - Fc map (blue) is contoured at 1.0 σ and the Fo - Fc map is contoured at 3.0 σ (green) and −3.0 σ (red). The GSSG molecule and neighbouring residues are shown as stick models and the solvent molecules are shown as red stars. (**b**) The key interactions of the GSSG molecule in the FhGST-S1 active site shown using a LIGPLOT+ diagram^35^.

**Figure 5:**
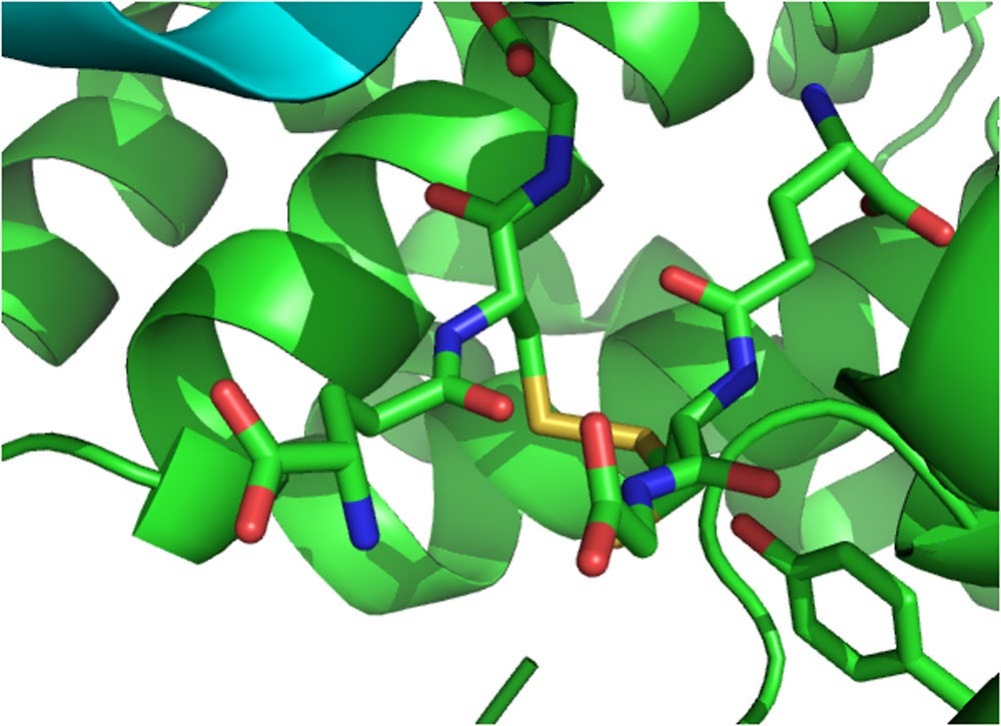
Binding of the GSSG complex to the GST G-site. Two monomers of FhGST-S1 are shown as green and cyan cartoon models. Two GSH molecules linked by a disulfide bond to form GSSG and the interacting Tyr10 residue are shown as stick models.

Clear density was observed for the glutathione in each monomer of each structure and was modelled as such. Additional density was observed in subunit A of the holo-structure, continuous from the Sγ of the glutathione moiety and extending to the Tyr106 OH. The additional density has been successfully modelled with cysteine covalently attached to the glutathione by a disulfide bond (cystine glutathione, CGL; Fig. 3). Additional density was also observed in each subunit of the GSSG-structure, continuous from the Sγ of the glutathione. In both subunits this extra density has been successfully modelled as a further glutathione molecule (Fig. 4) at partial (0.7) occupancy, therefore resulting in a mixture of GSH and glutathione disulfide present in the active site (Fig. 5).

### The G-Site

Subunit B of the holo-structure has only GSH bound, which is bound in the extended conformation and forms salt bridges with Arg16, Lys45 and Glu70 from the N-terminal domain of the same chain, and with Asp104 from the C-terminal domain of the other monomer chain. It also has hydrogen bonds to three main chain atoms (Val 53 N and O, Ser 71 N) and three hydrogen bonds to amino acid side chain atoms (Tyr 10 OH, Trp 41 NE2, Ser 71 OG). The Sγ atom of the GSH is modelled in two positions, A and B. In position A this atom is at a distance of 4.6 Å from the Tyr10 OH, and 2.77 Å from a water molecule (HOH 2155). In the alternative conformation, position B, the Sγ atom is 3.1 Å from the Tyr10 OH atom and 2.9 Å from the same water molecule. The interactions of GSH are the same as those above in both subunit A of the holo-structure and in both subunits of the GSSG-structure, with the exception of the distance of the Tyr10 OH atom to the Sγ atoms of the CGL and GSSG complexes. In the CGL complex the Tyr10 OH is 2.89 Å from the Sγ atom position of the free GSH and 4.15 Å from the Sγ atom in the CGL complex. There are no water molecules closer than 4.9 Å. In the GSSG complex subunits the Tyr10 OH atom is closer to the Sγ atoms of the GSSG complex than the Sγ atom of the GSH. In subunit A Tyr10 OH is at a distance of 3.35 Å and in subunit B 3.19 Å from the Sγ atom of the GSSG complex. In the case of the GSH, the distances are 4.25 Å for subunit A and 4.45 Å for subunit B and again there are no water molecules closer than 3.6 Å. The altered position of the Tyr10 side chain may be influenced by the presence of a DMSO molecule in the active site of each subunit. The oxygen atom of the DMSO in subunit A (DMS 1214) is 2.67 Å from the Tyr10 OH, and in subunit B (DMS 1212) 2.37 Å from the OH group.

### The H Site

The substrate binding site of the GST enzymes is termed the ‘hydrophobic’ site, or the H site. The residues involved in formation of the H-site are less conserved in the GST super-family, varying greatly between GSTs from the same class. There is a cleft in the surface of the protein leading towards the Sγ atom of the GSH moiety. This surface extends to form a cavity leading through the GST dimer to the second active site. Residues forming this surface cleft and cavity come from both the N-terminal domain (Tyr10, Phe11, Phe13 Arg16, Met38) and from the C-terminal domain (Glu103, Asp104, Tyr106, Arg107, Tyr110, Phe113, Arg114). The amino acid side chains of the Arg107 residues from both subunits form the lid to the channel between the active sites. Both of these amino acids were found to have two side chain conformations, indicating that their position can alter the shape of the cavity. The Met38 also adopts two side chain conformations in the structure, making changes to the shape of the surface cleft leading to the active site. Calculation of the electrostatic surface potential shows that the external cleft of the H-site is hydrophobic, with a positively charged surface accommodating the COOH group of the CGL ligand (holoenzyme structure subunit A), beyond which the surface loses charge.

### The CGL complex

The disulfide bound cysteine is only modelled at half occupancy, and an alternative position for the GSH Sγ atom is seen. The free Sγ atom position (Sγ B) is at a distance of 3 Å from the Tyr10 OH atom. The ligated cysteine makes an additional two hydrogen bonds to amino acid side chains, one to the Tyr10 OH atom, and one to the Tyr106 OH atom, and a further hydrogen bond to the main chain N atom of Arg16. There are further non-bonded contributions to the binding from the amino acid residues Phe11, Phe13, Gly15, Pro54 and Tyr110.

### The GSSG complex

The second glutathione molecule bound to both active sites of the GSSG-structure does not occupy the same site as the cysteine ligand in the CGL complex. The second glutathione makes few direct interactions, forming bonds to Arg 52 of the same subunit and Glu 108 of the other subunit. Non-binding interactions are made from the side chains of amino acids Tyr 110 from the same subunit and Asn 131 and Leu 135 from the other. No interactions are made from the half of the molecule that protrudes away from the active site, and this part of the glutathione is seen to occupy different spaces in each of the monomers.

### Comparison of holo, GSSG-structures and CGL complex

**Figure 6:**
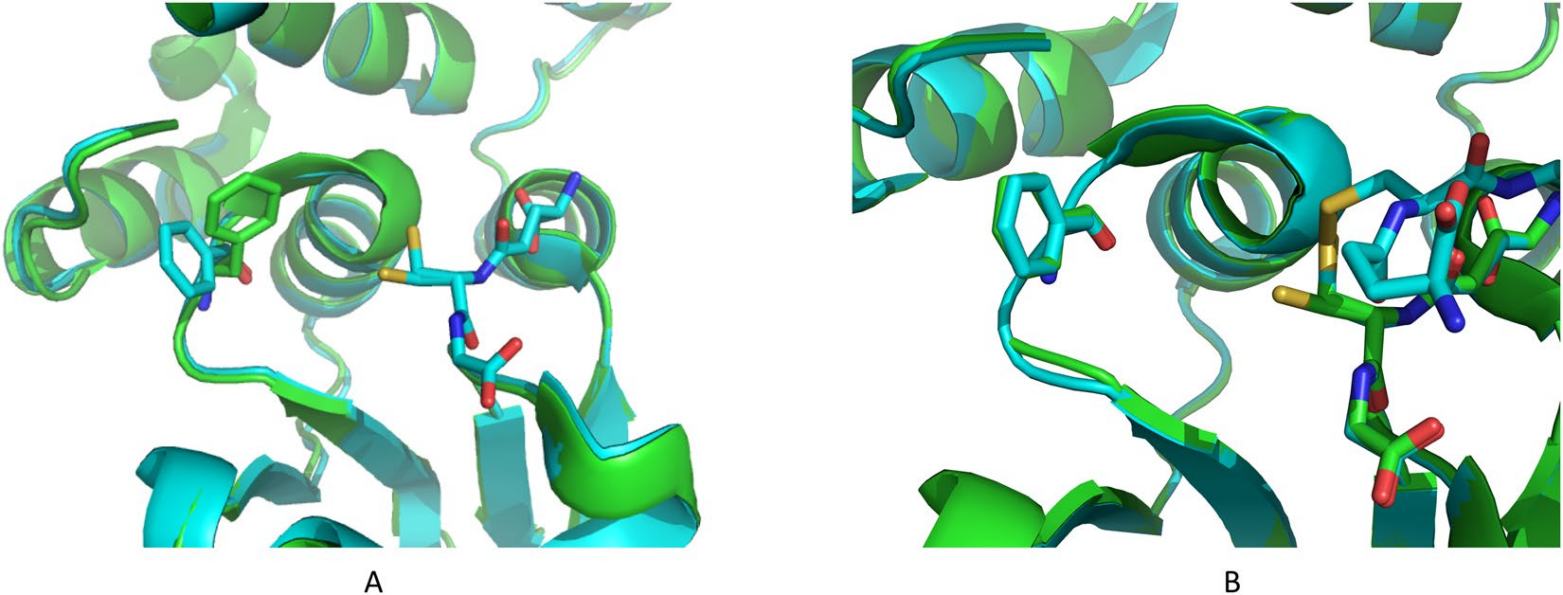
The movement of residue Phe13 caused by free cysteine residue binding to GSH in the FhGST-S1 G-site. (**A**) Cartoon models of the superimposition of the holo GST structure (cyan) and the CGL (green) complex structure. The Phe 13 side chain moves towards the active site upon formation of the CGL (GSH and cysteine) complex. The GSH molecule is shown as a stick model. The CGL molecule is not shown. (**B**) Superposition of cartoon models of the GST holo structure (green) and the GSSG complex (cyan). The position of the Phe13 side chain does not change from that observed in the holo structure upon GSSG binding.

Superposition of the two subunits of the holo-structure reveals that there are very few changes in response to the binding of the cysteine ligand. There is a small movement of the Tyr10 side chain, resulting in a movement of 0.7 Å between the OH atom positions. The residue Phe13 has a significant side chain movement (Fig. 6). Phe13 in subunit A has moved closer to the active site with a distance between the two Cβ positions of 0.9 Å. The side chain has rotated about Cβ by 70°. This residue is involved in non-bonded contacts to the cysteine ligand. Superposition of the holo-structure and the GSSG-structure (RMSD 0.28) reveals that the Phe13 residue occupies the same position as that found in the holo structure (Fig. 6). There are few changes between the structures, except around the position of the disulfide bond, where there are local changes to the disulfide bond position and the position of the surrounding Cα-backbone.

### Structural comparisons

The FhGST-S1 structure was submitted to DALI^19^ for comparison to other known structures. The top hit, (with a Z-score of 29.7, RMSD 1.5 Å) is the structure of the Sigma class GST from *Schistosoma haematobium* (pdb 2C80; Sh28GST). The studies of the *S. haematobium* enzyme revealed that the active site tyrosine residue adopted two side chain positions termed ‘in’ and ‘out’ depending on whether they pointed in towards the GSH thiol group or not^20^. The studies of these authors examined the data of a number of other GST structures and found that these structures also contained additional density and space for the ‘out’ position of the Tyr residue. In the structures reported here, there is no additional density for the alternative ‘out’ position of Tyr10 and in fact, very little movement is observed between the two monomers. The authors of these studies found Arg35 was highly conserved and was found in 68% of Alpha, Mu, Pi and Sigma GST sequences also containing a catalytic Tyr residue using a query motif [Y] 7X [E, Q, H] 2X [R] 10–11X [E, D] 1–2X [R, K]. The conserved Arg35 was found to take two alternative side chain positions dependent on the ‘in’ or ‘out’ position of the catalytic Tyr residue and to interact with Asp33 closing the Tyr^out^ pocket^21^. In FhGST-S1, whilst Asp33 is conserved, Arg35 is not and is found to be a glutamine (Gln35), therefore not conserving this interaction.

**Figure 7:**
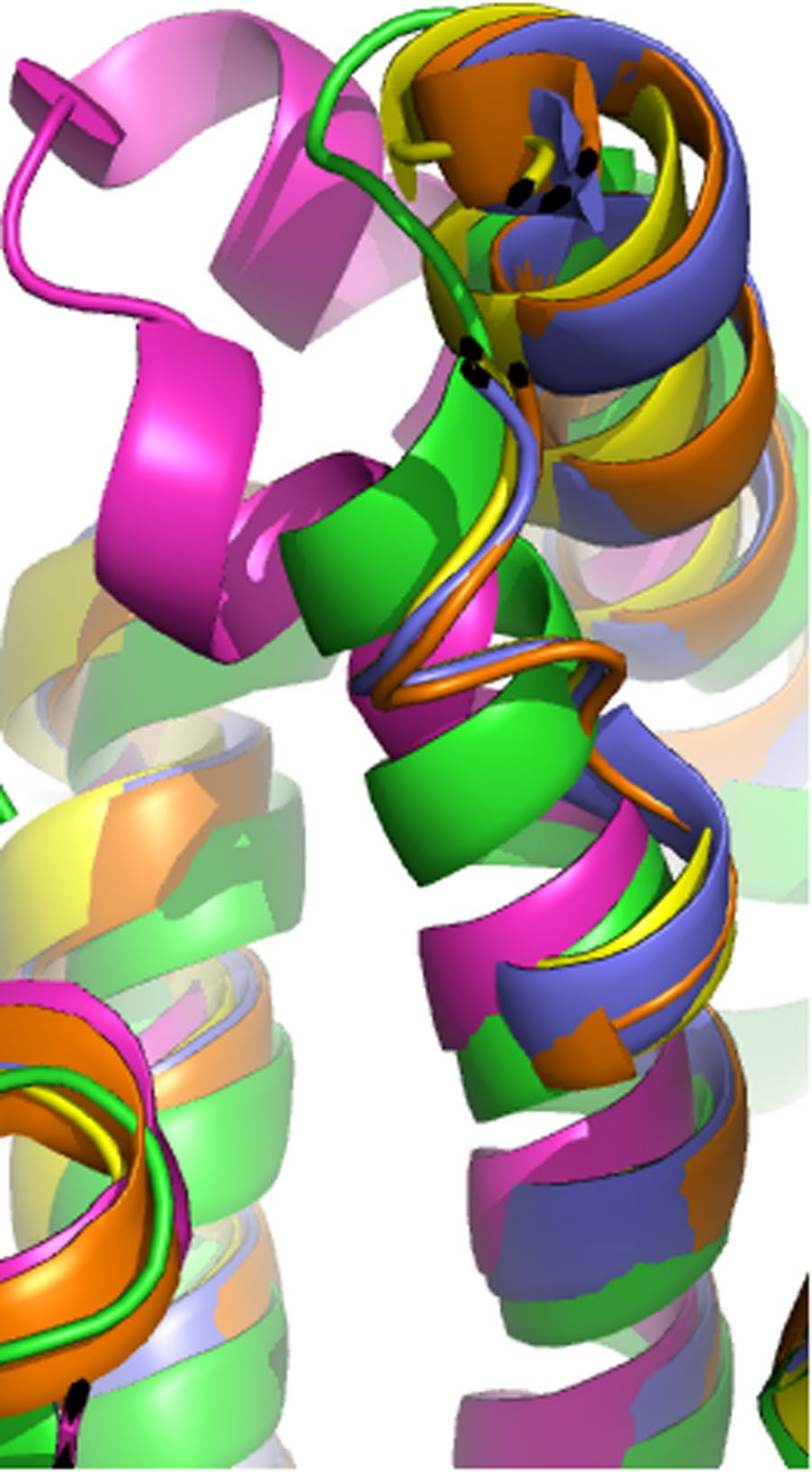
Superposition of helix α4 of FhGST-S1 (green), *O. volvulus* GST-1 (pink, pdb 2HNL), and PGDS from *Rattus norvegicus* (yellow, pdb 1pd2), and *Homo sapiens* (blue, pdb 1IYI; orange, pdb 3EE2). The FhGST-S1 helix is not as long as that from *O. volvulus* GST, and aligns better to the equivalent mammalian helix.

The structure of an extracellular Sigma class GST from the human filarial nematode parasite *Onchocerca volvulus* (*Ov*GST1 – pdb 2HNL) has previously been reported^22^. FhGST-S1 superposes to the *Ov*GST1 with an RMSD of 1.95 Å across 192 residues. The *Ov*GST1 has also been shown to exhibit PGDS activity and is thus implicated in immune response modulation^22^. Interestingly, the nematode enzyme shows a higher sequence identity to mammalian PGDS than to other parasitic worm Sigma class GSTs^22^. Although *Ov*GST1 is a Sigma class GST, the structure revealed it to have two ‘lock and key’ interactions at the dimer interface, one of which is akin to that found in human haematopoietic PGDS^22,23^. This feature is not observed in the *F. hepatica* GST-S1. However, the prostaglandin binding site of the *Ov*GST1 was found to differ considerably to those of mammalian PGDS proteins^22^, suggesting a different mode of binding is required for the filarial enzyme. PGDSs have a conserved Trp residue in the loop between α4 and α5, causing a backbone kink, resulting in the shortening of helix α4. Like the *Ov*GST1, FhGST-S1 does not have this Trp residue and therefore has a longer α4, although the FhGST-S1 helix aligns better to that of the mammalian PGDSs (Fig. 7). Again like the *Ov*GST1, FhGST-S1 has little conservation of the proposed prostaglandin binding residues, suggesting as with the *Ov*GST1, a different mode of prostaglandin binding. Of the residues proposed to be involved in prostaglandin binding in *Ov*GST1^22^, only three are the same amino acid in the equivalent positions in FhGST-S1. As such, it is difficult to currently ascertain if the FhGST-S1 is acting in the same way as the *Ov*GST1.

The highest sequence identity human GST counterpart is a PGDS (pdb 3EE2). FhGST-S1 superposes to this enzyme with an RMSD of 1.91 Å over 192 Cα atoms. Interestingly, with respect to selectivity and potential drug development, FhGST-S1 demonstrated a different inhibition profile to hGSTS1–1 (PGDS) when probed with a series of isoquinolyl inhibitors that were synthesised as previously described^24^ (Supplementary Table 1).

### Conclusions

The *Fasciola* Sigma class GST, FhGST-S1 has been shown to have the same fold as other GSTs and to be highly similar to other Sigma class GSTs. However, the protein was shown to have an interdomain disulfide bond, which has not been observed in any GST structures previously described. The relationship of the two domains with respect to each other is crucial in the formation of the active site cleft and therefore the presence of a disulfide bond to stabilise this interaction is of great interest. The enzyme is found in fluke extracellular vesicles and this indicates that it does not require a classical leader sequence to leave parasite tissues. If the Sigma GST FhGST-S1 is then pro-actively released into the host gastro-intestinal environment via EVs then the presence of the disulfide bond would stabilise the released protein in its non-reducing host environment.

## Materials and Methods

### Expression and purification

The FhGST-S1 was cloned into pET23a (Novagen) as described previously^11^. The recombinant protein was expressed in *E. coli* BL21 (DE3) cells (Novagen). Initial cultures were grown overnight at 37 °C in LB supplemented with 100 μg/ml ampicillin. The Initial culture was used to inoculate a 1 L culture (LB, 100 μg/ml ampicillin) diluted down to an OD_600_ of 0.05–0.1. This culture was grown for a further 5 hours at 37 °C shaking at 200 rpm. The culture was transferred to 28 °C and allowed to equilibrate for 30 minutes before the addition of IPTG to a final concentration of 0.5 mM. The culture remained at 28 °C for a further 2 hours before cells were harvested by centrifugation (8000 × *g*, 20 minutes, 4 °C). Cell pellets were resuspended in 50 mM Tris-HCl, pH 8.0 and cells disrupted by sonication (SoniPrep Ultrasonic Disintegrator, Sanyo) with incubations on ice between sonication bursts. Cell debris was removed by centrifugation (12000 × *g*, 20 minutes, 4 °C). The clarified extract was applied to a pre-swollen glutathione agarose column (Sigma-Aldrich) equilibrated with 20 mM potassium phosphate pH 7.0. The glutathione agarose column was washed with equilibration buffer and eluted using 5 mM glutathione in 50 mM Tris pH 8.0. Elution fractions containing FhGST-S1 were pooled and concentrated using a centrifugal concentrator (PES membrane, 10 kDa MWCO, Sartorius) and applied to a pre-equilibrated Superdex 200 column (G.E. Healthcare) and eluted with 100 mM Tris-HCl, pH 7.5. The recombinant FhGST-S1 enzyme was shown to be active, following the conjugation of substrates 1-chloro-2,4-dinitrobenzene (CDNB) and reduced glutathione according to an adaptation of the method described previously^25^.

### Crystallisation and data collection

Purified FhGST-S1 was concentrated to 10 mg/ml using a centrifugal concentrator (PES membrane, 10 kDa MWCO, Sartorius) at 4 °C and frozen in aliquots. 10 μl of 100 mM glutathione and 10 μl 100 mM TCBZ in DMSO was added to 380 μl of protein. The sample was centrifuged for 5 minutes to clarify the solution. Crystallisation screening was carried out using an Oryx 6 crystallisation robot (Douglas Instruments) in microbatch mode. The crystals were grown from condition G10 of JCSG-*plus*^26^, Molecular Dimensions Ltd), 0.15 M KBr, 20% (w/v) PEG 3350. Multiple crystals grew in clusters and these were cut whilst in the droplet to obtain a single crystal. For flash freezing the crystal was harvested into a cryogenic liquor containing 100 mM Tris-HCl pH 7.5, 0.15 M KBr, 17% (w/v) PEG 3350, 25% (w/v) PEG 400. Data were collected in house at 100° K on a MAR Research 345 Image Plate mounted on a Siemens rotating anode generator using copper Kα radiation and XENOCS FOX2D CU_25P mirrors. An initial data set was collected to 2.0 Å resolution, and the background diffraction from other contaminating crystal lattices was found to be minimal and did not interfere with the data processing. A further data set was collected to 1.6 Å from the same crystal and was used for the structure solution. Structure solution of this data set revealed a cysteine ligand bound in the active site.

Further crystals were grown from protein concentrated to 20 mg/ml with the addition of 0.5 μl of 100 mM glutathione and 4 μl 1 M TCBZ in DMSO added to 40 μl of protein. The sample was centrifuged to clarify the solution as before. Crystals were harvested into the cryogenic mother liquor described above, with the inclusion of 100 mM TCBZ in DMSO. Again the diffraction data revealed the crystals to contain more than one lattice, but this was not detrimental to data processing. Data were collected in house to 1.6 Å resolution. Solution of this data revealed the presence of glutathione disulfide GSSG molecule bound in the active site.

### Structure solution, model building and refinement

Data were processed with MOSFLM^27^ and SCALA^28^ using the CCP4 program suite^29^. The FhGST-S1 structure was solved by molecular replacement using the program MOLREP^30^ with the *S. mansoni* 28 kDa GST structure (1U3I) as the model. Refinement was carried out using REFMAC 5.2^31^ and model building was carried out using COOT^32^. Water molecules were added using ARP/wARP^33^. Images were created using the molecular graphics program PyMol^34^.

### Data delivery

The structure factors and the refined coordinates of the FhGST-S1 structures have been deposited with the Protein Data Bank and have the access codes 2WB9 and 2WDU.

## Supplementary information

Dataset 1
