# Supplementary material for: X-ray structure of Fasciola hepatica Sigma class glutathione transferase 1 reveals a disulfide bond to support stability in gastro-intestinal environment
Source: Sci Rep. 2019 Jan 29;9:902. doi: 10.1038/s41598-018-37531-5 (PMC6351632; doi:10.1038/s41598-018-37531-5)
Supplement: Supplementary file 1 — Dataset 1 [file 41598_2018_37531_MOESM1_ESM.pdf]

Supplementary Table 1 SREP-18-33649A

X-ray structure of *Fasciola hepatica* Sigma class glutathione transferase 1 reveals a disulfide bond to support stability in gastro-intestinal environment

Kirsty Line[1], Michail Isupov[1], E. James LaCourse[3], David J. Cutress[2], Russell M. Morphew[2], Peter M. Brophy[2*] and Jennifer A. Littlechild[1]

Affiliations

[1]Henry Wellcome Building for Biocatalysis, Biosciences, College of Life and Environmental Sciences, University of Exeter, Stocker Road, Exeter, EX4 4QD, UK

[2]Institute of Environmental and Rural Sciences (IBERS), Aberystwyth University, SY23 2DA, UK

[3] Liverpool School of Tropical Medicine, Liverpool, Pembroke Place, L3 5QA, UK

* Corresponding Author Email: pmb@aber.ac.uk
Telephone +44(0)1970 622332

Supplementary Table 1
Evidence of selective inhibition between recombinant *Fasciola hepatica* Sigma GST-1 (FhGST-1) and *Fasciola gigantica* GST-1 (FgGST-1) and equivalent human enzyme hGSTS1-1 (PGDS)

Inhibitors synthesised as numbered in Trujillo et al. Bio & Med. Chem. Lett. 22, 3795	IC50 μM		Published IC50 μM
	FhGST-S1	FgGST-S1	hGSTS1-1 (PGDS)
Inhibitor C9	457.22	508.14	0.00234
Inhibitor C10	514.67	742.52	0.274
Inhibitor C12	171.52	421.51	0.662
